# Real-time EEG-based brain-computer interface to a virtual avatar enhances cortical involvement in human treadmill walking

**DOI:** 10.1038/s41598-017-09187-0

**Published:** 2017-08-21

**Authors:** Trieu Phat Luu, Sho Nakagome, Yongtian He, Jose L. Contreras-Vidal

**Affiliations:** Noninvasive Brain-Machine Interface System Laboratory, Dept. of Electrical and Computer Engineering, University of Houston, Houston, TX 77004 USA

## Abstract

Recent advances in non-invasive brain-computer interface (BCI) technologies have shown the feasibility of neural decoding for both users’ gait intent and continuous kinematics. However, the dynamics of cortical involvement in human upright walking with a closed-loop BCI has not been investigated. This study aims to investigate the changes of cortical involvement in human treadmill walking with and without BCI control of a walking avatar. Source localization revealed significant differences in cortical network activity between walking with and without closed-loop BCI control. Our results showed sustained α/µ suppression in the Posterior Parietal Cortex and Inferior Parietal Lobe, indicating increases of cortical involvement during walking with BCI control. We also observed significant increased activity of the Anterior Cingulate Cortex (ACC) in the low frequency band suggesting the presence of a cortical network involved in error monitoring and motor learning. Additionally, the presence of low γ modulations in the ACC and Superior Temporal Gyrus may associate with increases of voluntary control of human gait. This work is a further step toward the development of a novel training paradigm for improving the efficacy of rehabilitation in a top-down approach.

## Introduction

Non-invasive scalp electroencephalogram (EEG) has recently been used to monitor cortical activities during human walking because of its portability and high time resolution^1, 2^. Prior studies suggested that EEG recording during walking display features that differ from those reported during standing, and more importantly, is coupled with the gait cycle^1, 3–5^. Electrocortical activities have also been used to distinguish between uphill versus level walking in humans^6^ and active versus passive walking in a robotic device^7^. A study with an active treadmill demonstrated that user-driven control increases cortical activity^8^.

These findings provide evidence that EEG has the capacity to monitor cortical activity in treadmill walking, enabling EEG-based Brain-Computer Interface (BCI) systems for walking. BCIs are systems that allow brain activity to directly control physical or virtual robots and machines. Non-invasive EEG-based BCIs have been proposed to control hand^9, 10^ and arm^11^ robotic devices. BCIs for the lower-limbs were proposed in offline analysis^12–15^, and validated in a discrete online control of an exoskeleton^16^ and a walking avatar in virtual reality (VR)^17^. With recent advances of VR devices, VR has been proposed as an alternative tool in stroke rehabilitation^18^. VR has been explored in a few walking-related BCI systems^19–21^. It provides real-time feedback to BCI users, while avoiding many technical challenges that lower-limb robotics face, such as high cost, risk of falls, and joint misalignment.

Recently, our group has successfully reconstructed human gait kinematics in real-time from EEG signals^21, 22^. In our previous study, we designed a real-time neural decoder to predict lower limb kinematics of human treadmill walking from EEG signals. The predicted joint angles (hip, knee, and ankle) were used to control a walking avatar in a virtual environment^21^. We demonstrated that BCI performance increased across sessions in multiple days^21^. However, to our best knowledge, the dynamics of cortical involvement in human upright walking with a closed-loop BCI has not been investigated. This study aims to investigate the changes of cortical involvement in human treadmill walking with and without BCI control of a walking avatar. Source localization using independent component analysis revealed significant differences in cortical network activity between walking with and without closed-loop BCI control. We hypothesized that the closed-loop EEG-based BCI-VR system enhances cortical involvement in human treadmill walking, triggers cortical networks involved in error monitoring and motor learning, and increases voluntary control of human gait. Once confirmed, it will have important implications in transferring BCI-based protocols to gait rehabilitation.

## Materials and Methods

### Experimental setup and procedure

**Figure 1:**
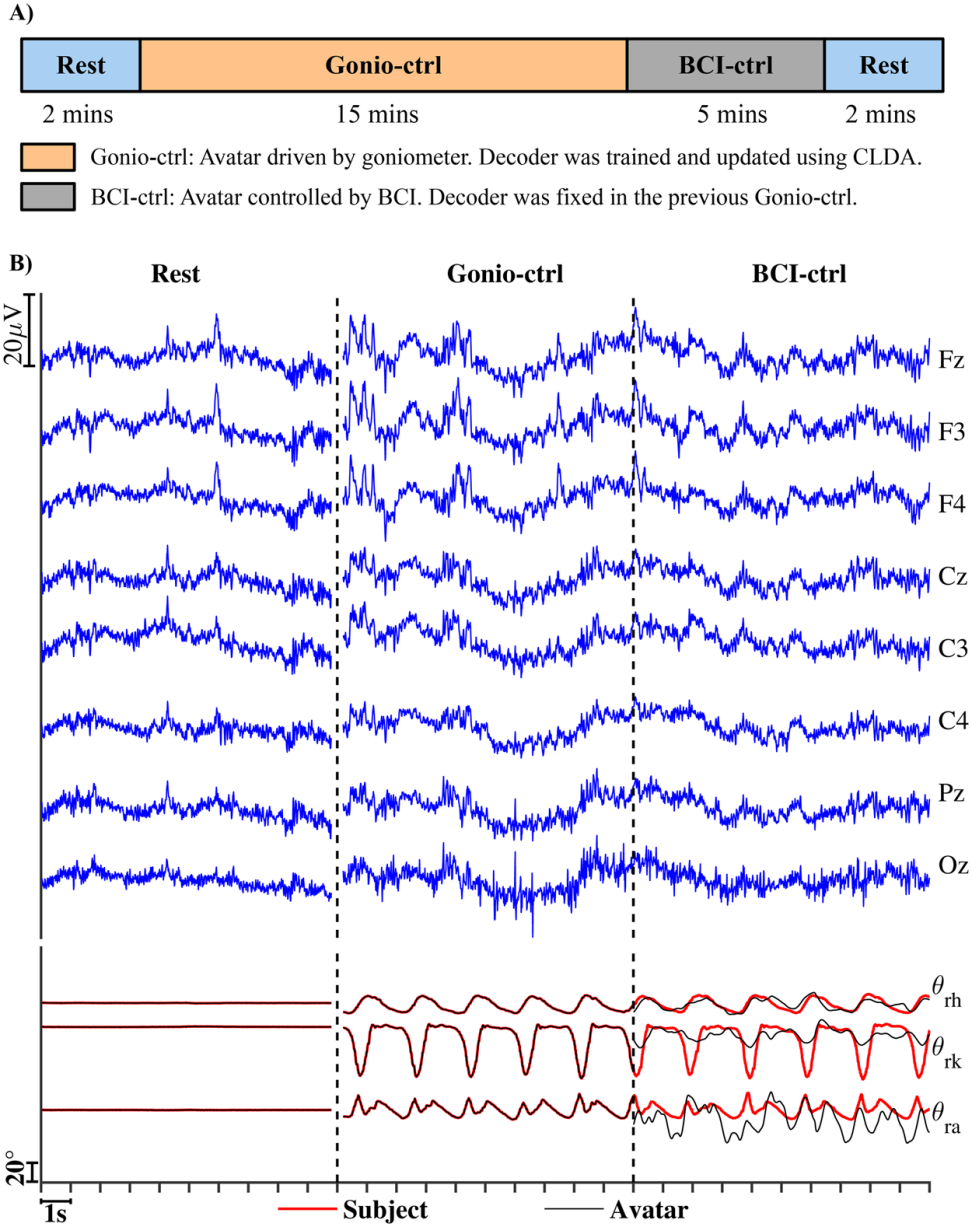
The experimental protocol, raw EEG, and examples of predicted and actual joint angles in this study. (**A**) Experimental protocol for each trial. In the Gonio-ctrl phase (15 mins), the avatar was driven by goniometer signals and the decoder’s parameters were updated every one min by using a CLDA algorithm. In the BCI-ctrl phase (5 mins), the decoder’s parameters were fixed and the avatar’s right leg was driven by the outputs from the neural decoder (**B**) Example of raw EEG signals in each phase, actual (Subject, red lines), and predicted (Avatar, black lines) lower limb joint angles.

Eight healthy individuals (three males, five females; aged from 19 to 29) with no history of neurological disease or lower limb pathology participated in this study. All experimental protocols and informed consent (signed by all participants) were approved by the Institutional Review Board (IRB) at the University of Houston. All experiments were performed in accordance with the 45 Code of Federal Regulations (CFR) part 46 (“The Common Rule”), specifically addressing the protection of human study subjects as promulgated by the U.S. Department of Health and Human Services (DHHS). Written informed consent to publish identifying images was also obtained. Each subject participated in two sessions with a total of three trials (two consecutive days, two trials in the first day). The subjects were instructed to have two minutes (mins) of standing still on a treadmill in the beginning and end of each trial. In the remaining period, the subjects walked on a treadmill at one mile per hour (mph) while looking at an avatar displayed eye-level on a 52-inch TV monitor placed in front of the treadmill. The movements of the avatar’s left leg in the sagittal plane followed the subjects’ movements precisely all the time by using the data from goniometer sensor placed at hip, knee, and ankle joint angle. However, the avatar’s right leg was driven either by goniometer sensors or predicted joint angles from BCI system depending on the protocol (Fig. 1). For safety purposes, all subjects were instructed to hold onto a front handle bar while walking on the treadmill.

Figure 1 shows the experimental protocol, EEG signals, and the subject’s and avatar’s joint angles. Each trial began and terminated with the subject standing still on the treadmill for two mins while looking at the avatar displayed on a monitor. In the rest phase, the avatar was standing and it was controlled by goniometer sensors to follow the subjects’ movements precisely. The rest phase was included because data in this phase were used as baseline for artifact subspace reconstruction (ASR) in EEG offline processing. Moreover, it provided continuous movements of the avatar from standing still, increasing the speed, and walking on a treadmill. Subjects were asked to minimize his/her eye blinks in the rest phase. After the first two mins, the treadmill speed was then increased to one mph. In the *Gonio-ctrl* phase (15 mins), the walking avatar was driven by signals from goniometer sensors and its movement followed the subject’s movement correctly. During this phase, the neural decoder (unscented Kalman filter, UKF) and its parameters were trained and updated in the background every minute by using a closed-loop decoder adaptation algorithm (CLDA). Details of CLDA-UKF decoder can be found in refs 17, 21. We found that the UKF’s parameters had to be updated about 15 times (15-min period) to yield the best decoding accuracy of lower limb joint angles. At the end of the *Gonio-ctrl* phase, the UKF’s parameters were fixed and the protocol switched to *BCI-ctrl* phase. In the *BCI-ctrl* phase (5 mins, operated at 10 ms cycle time), the avatar’s right leg was driven by the predicted joint angles from the UKF neural decoder. We developed the unilateral BCI-VR system to control only the right leg of the avatar for the following reasons: 1) The system will be used as a platform to examine the changes of neural representations for action and perception of bipedal locomotion during adaptation to virtual cortical lesions in one hemisphere, 2) Controlling both legs of the walking avatar from closed-loop BCI could be challenging to the subjects and they might also loose the interlimb phasing if the neural decoder was not perfect, and 3) A unilateral closed-loop BCI system could enhance cortical differences across conditions.

### Data collection and signal processing for real-time BCI operations

The processing for both EEG and kinematic data for real-time closed-loop BCI was described in detail in our previous study^21^ and summarized here for completeness. All the real-time processing was run in our custom C++ software. First, we used data from EOG channels and run a robust adaptive filter to remove eye related artifacts (eye blinks and eye movements)^23^. Peripheral EEG channels were not used in the real-time decoding because they are most susceptible to artifacts from head movements and facial/cranical muscle activity. We used the fluctuations in the amplitude of slow cortical potential in the delta band (0.1–3 Hz) to decode lower limb joint angles. Lower limb joint angles were also low-passed filtered at 3 Hz using a Butterworth filter. This band was known to cover most power in the lower limb joint angle signals^24^. We used the fluctuations in the amplitude of slow cortical potential in the delta band (0.1–3 Hz) to decode lower limb joint angles. An Unscented Kalman filter (UKF) was implemented as neural decoder and its parameters were updated during BCI operations by using a closed-loop decoder adaptation (CLDA) to improve the performance^17, 21^.

Whole scalp 64-channel active EEG data were collected (ActiCap system, Brain Products GmbH, Germany) and labeled in accordance with the extended 10–20 international system. A wireless interface (MOVE system, Brain Products GmbH, Germany) was used to transmit data (sampled at 100 Hz) to the host PC. The channel layout was modified from the standard EEG cap setup. Ground (GND) and reference (REF) channels were placed on the left and right earlobe (A1 and A2), respectively. T7 and T8 channels were moved to FCz and AFz, respectively. FT9, FT10, TP9 and TP10 were used for electrooculography (EOG) to capture eye blinks and eye movements. The purpose of channel layout modification is to improve the real-time decoding accuracies based on our previous results. The two main reasons for the modification are: 1) GND and REF channels in the standard EEG cap layout are very close to the motor cortex which has been known to be important for neural decoding of human walking, and 2) EOG sensors were required in our real-time artifact removal algorithm^21^. Lower limb joint angles (hip, knee, and ankle) in the sagittal plane were recorded by goniometers (SG150 & SG110/A Gonio electrodes, Biometrics Ltd, UK) at 100 Hz, and in sync with EEG data using our customized C++ program. Details for the experimental setup can be found in the supplementary material and in our previous study^21^. Additionally, a 3D electrode localization system (BrainVision Captrak, Brain Products GmbH, Germany) was used to record electrode positions and consistently fit the cap to the subjects head across multiple sessions.

### Offline EEG Signal processing

**Figure 2:**
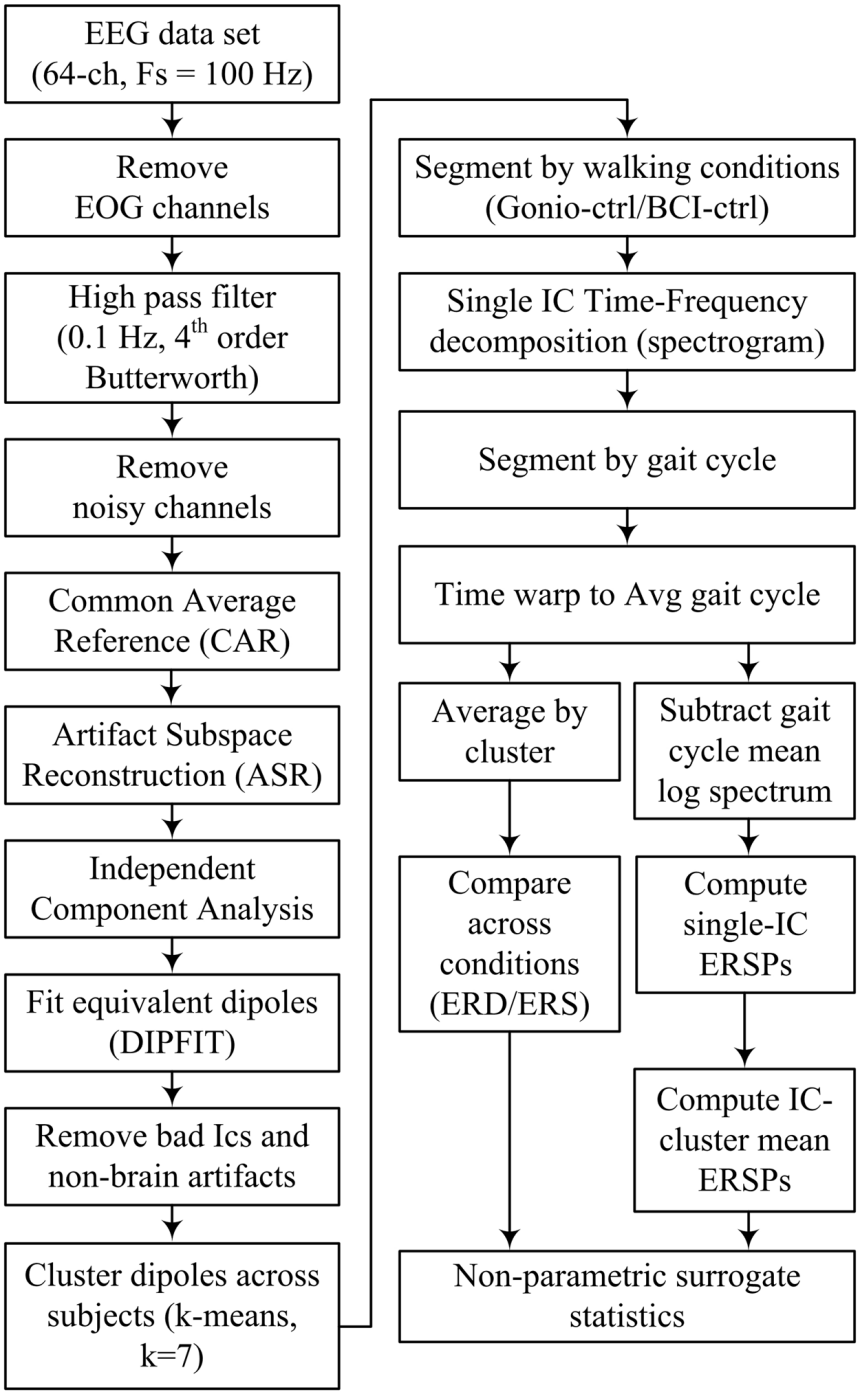
Flow-chart indicating EEG data-processing pipeline.

Data analysis and statistical analysis were performed using custom software written in Matlab R2016a (The MathWorks, Natick, MA.) and functions from EEGLAB v.13^25^. 64-ch EEG was recorded at 100 Hz and the EEG signal processing pipeline is illustrated in Fig. 2. Electrooculography (EOG) channels were first removed and the remaining EEG signals (60 channels) were high pass filtered at 0.1 Hz using a 4^th^ order Butterworth filter. Bad EEG channels, indicated as standard deviation greater than 1000 µV or kurtosis of more than five standard deviations from the mean, were rejected^3^. The remaining EEG channels were then re-referenced to their common average. Next, artifact subspace reconstruction (ASR) was applied to remove high amplitude artifacts (e.g., eye blinks, muscle burst)^26, 27^. ASR applies principal component analysis to the EEG data in sliding windows and identifies channels that significantly deviate from the baseline data containing minimal movement artifact. Channels with variance above a predefined threshold compared to the baseline data are identified as corrupted and reconstructed using a mixing matrix that is computed from the baseline data. Here, we implemented the ASR algorithm, which is available as a plugin for EEGLAB toolbox^25, 26^. In this study, one min of EEG recorded during quite standing at the beginning of each trial was used as baseline (calibration) data for the ASR. A sliding window (length of 500 ms) and a variance threshold of three standard deviations were used to identify corrupted subspaces.

An equivalent current dipole matched to the scalp projection of each independent component (IC) source was computed by using a standard three-shell boundary element head model included in the DIPFIT toolbox in EEGLAB^25^. We used a Captrak system (Brain Products GmbH, Germany) for scanning EEG electrode positions and aligned them to a standard MNI brain model (Montreal Neurological Institute, Quebec, Canada). Only ICs in which the equivalent dipoles were located within the brain volume and explained >90% of the residual variance of the IC scalp projections were retained for further analysis. Next, we visually inspected each IC scalp projection, and its power spectra and removed ICs corresponding to eye blink/movement and neck muscle artifacts. The remaining ICs were then used to generate feature vectors, which include the information of power spectral density (<50 Hz), topographical scalp projections, and dipole locations, and clustered across subjects using k-means algorithm (k = 7). ICs that were further than three standard deviations from a cluster centroid were categorized into an outlier cluster and omitted from further analysis.

Power spectral density (PSD) for each IC was computed using multitaper (seven Selpian tapers) fast Fourier transform. Cluster grand mean PSDs were then computed by averaging across the ICs within each cluster. A within subject, repeated-measures one-way ANOVA with Tukey-Kramer correction for post-hoc multiple comparison was used to assess the effects of different walking conditions (*Gonio-ctrl* and *BCI-ctrl*) on the area under PSD curve across four frequency band of interest: Δ (0.1–3 Hz), θ: 4–7 Hz, α/µ (8–13 Hz), β (14–30 Hz), and low γ (30–49 Hz). Note that only the last 5 minute of data from the *Gonio-ctrl* phase were used for further comparison to the *BCI-ctrl* phase. We also analyze the differences in time-frequency spectrograms between walking with and without BCI control. First, the time-frequency decomposition (spectrogram) was performed using a short-time Fourier transform for each IC in the clusters (Hamming window length of 500 ms, maximum overlap) across the whole trial. The full-length spectrogram was then segmented into gait cycles, which were detected from kinematic data, and linearly time-warped to the average gait cycle length so that right heel-strikes occurred at the same median latency^3^. Individual epoch spectrograms were concatenated for each cluster in each walking conditions. Finally, we computed the difference spectrograms by subtracting the two walking conditions. The difference spectrograms were masked for significance (α = 0.05) using a non-parametric bootstrapping technique with random shuffling of 200 surrogate data^25^.

To illustrate intra-stride modulations of power for each IC, gait cycle mean log spectrum in each epoch spectrogram was computed and subtracted from log spectrogram at each time point. The mean subtracted log spectrograms were then averaged across all gait cycles, resulting in spectral power changes relative to the average power over the gait cycle^3, 28^. These event-related spectral perturbations (ERSPs) were averaged across all ICs in a cluster to create grand mean ERSPs for each walking condition. Significant ERSP values were identified using a bootstrapping technique within EEGLAB (α = 0.05)^25^.

## Results

### Decoding performance of the closed-loop EEG-based brain computer interface (BCI)

**Figure 3:**
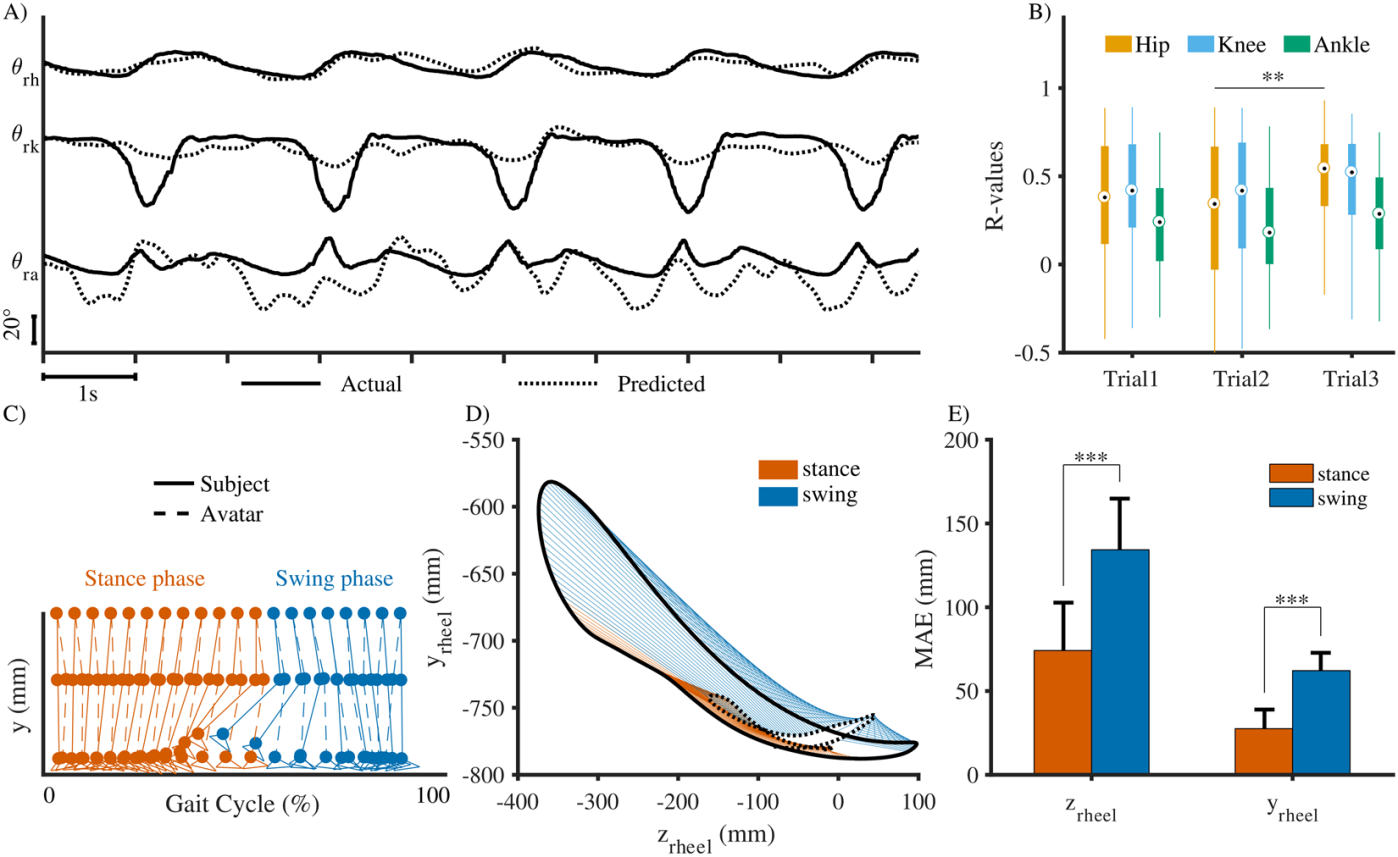
Closed-loop BCI control of the avatar resulted in visual-motor distortions. (**A**) Examples of reconstructed lower limb joint angles from EEG signals. R-value between the actual and predicted example data for the hip: 0.85; knee: 0.79; and ankle: 0.63. (**B**) Pearson’s r-values between the actual and predicted lower limb joint angles. In the box plots, the central rectangle spans the first to the third quartile, the center circle represents median r-value of each joint, and the whiskers above and below the box show the minimum and maximum r-values. (**C**) Illustration of subject’s and the avatar’s right leg during stance and swing phases. (**D**) Comparison between subject’s and the avatar’s heel position during stance and swing phases. (**E**) Mismatch between the subject’s and the avatar’s heel positions during stance and swing phases. *P < 0.05, **P < 0.01, and ***P < 0.001. MAE = Mean Absolute Error.

The performance of the real-time closed-loop neural decoder was evaluated by Pearson’s r-value between the measured and the predicted lower limb joint angles (Fig. 3). Overall, the median and interquartile range (IQR) of the r-values for all the subjects across all trials for the hip are (median: 0.42; IQR: 0.53), knee (median: 0.45; IQR: 0.49), and ankle (median: 0.28; IQR: 0.42). We found the best decoding accuracy with the median r-value for hip, knee, and ankle being 0.85, 0.79, and 0.63, respectively. Figure 3C–E illustrates the mismatch between the subject’s and the avatar’s right legs in one gait cycle. The errors were significantly higher in the swing phase as compared to the stance phase.

### Cortical IC source clusters and the features of their activations

**Figure 4:**
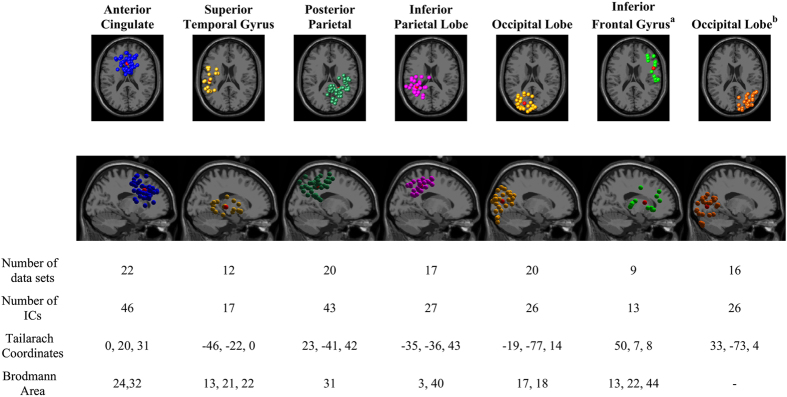
Clusters of dipolar sources fit to independent components for all subjects across all trials, which include *Gonio-ctrl* and *BCI-ctrl* of the avatar. Brodmann areas are the regions found within ±5 mm search range of cluster centroids. Seven cortical areas include Anterior Cingulate Cortex (ACC), Superior Temporal Gyrus (STG), Posterior Parietal Cortex (PPC), Inferior Parietal Lobe (IPL), Occipital Lobe (OL), Inferior Frontal Gyrus (IFG), and Right Occipital Lobe (OL) a) The IFG cluster was omitted from analysis because it did not contain ICs from a majority of data sets and b) Right Occipital Lobe is omitted because no Brodmann area was found for its cluster centroid.

Source localization using independent component analysis and k-mean clustering method revealed seven distinct cortical IC source clusters and one additional outlier cluster. Figure 4 shows the number of data sets and the number of IC sources contained in each cluster. Information of the cluster centroid such as the Talairach coordinates and Brodmann areas, which were identified from Talairach atlas^29^, is also illustrated. The Brodmann areas (BA) were searched within ±5 mm cube ranges around each cluster centroid. The searching resulted in 7 cortical areas such as: Anterior Cingulate Cortex (ACC; BA: 24, 32), Superior Temporal Gyrus (STG; BA: 13, 21, 22), Posterior Parietal Cortex (PPC; BA: 7, 31), Inferior Parietal Lobe (IPL; BA: 3, 40), Occipital Lobe (OL; BA: 17, 18), Inferior Frontal Gyrus (IFG, BA: 13, 22, 44), and Right Occipital Lobe (BA: -).

**Figure 5:**
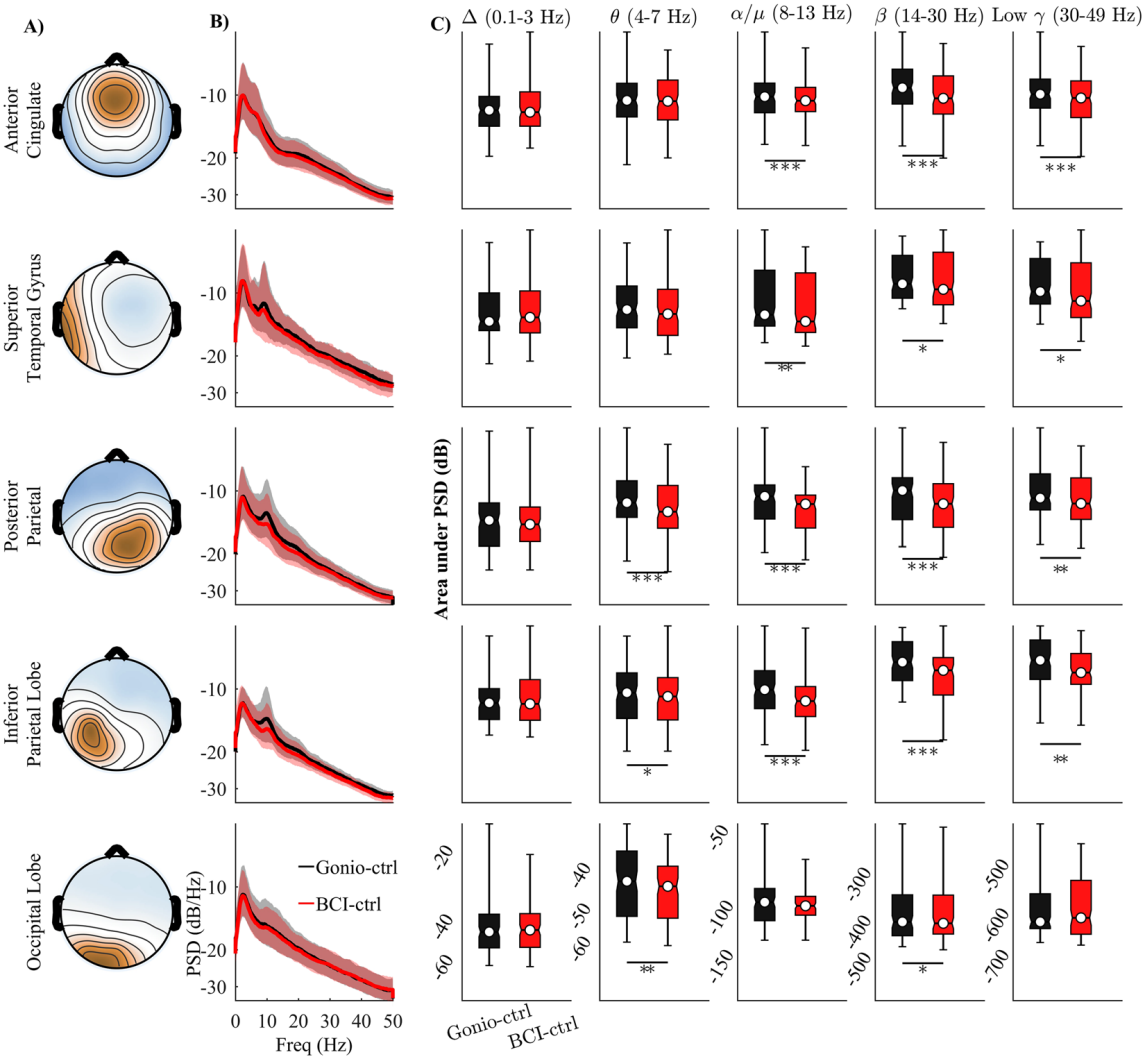
Scalp map and spectral characterization of task-related IC components. (**A**) The average of topographical scalp projections of independent components comprising the cortical clusters. (**B**) Average and standard deviation of power spectra density (PSD) across all ICs in each cluster for different walking conditions: *Gonio-ctrl* and *BCI-ctrl*. Solid line and shaded area represent the mean PSD curve and its standard deviation, respectively. C) Average of the area under PSD curve in different frequency bands. Significant level *P < 0.05, **P < 0.01, and ***P < 0.001.

Figure 5 shows the average power spectra density (PSD) across ICs for each cluster in different experimental conditions (*Gonio-ctrl* and *BCI-ctrl*). The areas under the PSD curves in different frequency bands (Δ: 0.1–3 Hz; θ: 4–7 Hz; α/µ: 8–13 Hz; β: 14–30 Hz; and low γ: 30–49 Hz) were also computed. The results were averaged across clusters and statistical comparison across conditions was also performed and showed in Fig. 5. Post-hoc comparison reveals significant decreases of spectral power in the α/µ, β, and low γ bands in the *BCI-ctrl* as compared to the *Gonio-ctrl* condition. The differences occur in all clusters except for the Occipital Lobe.

**Figure 6:**
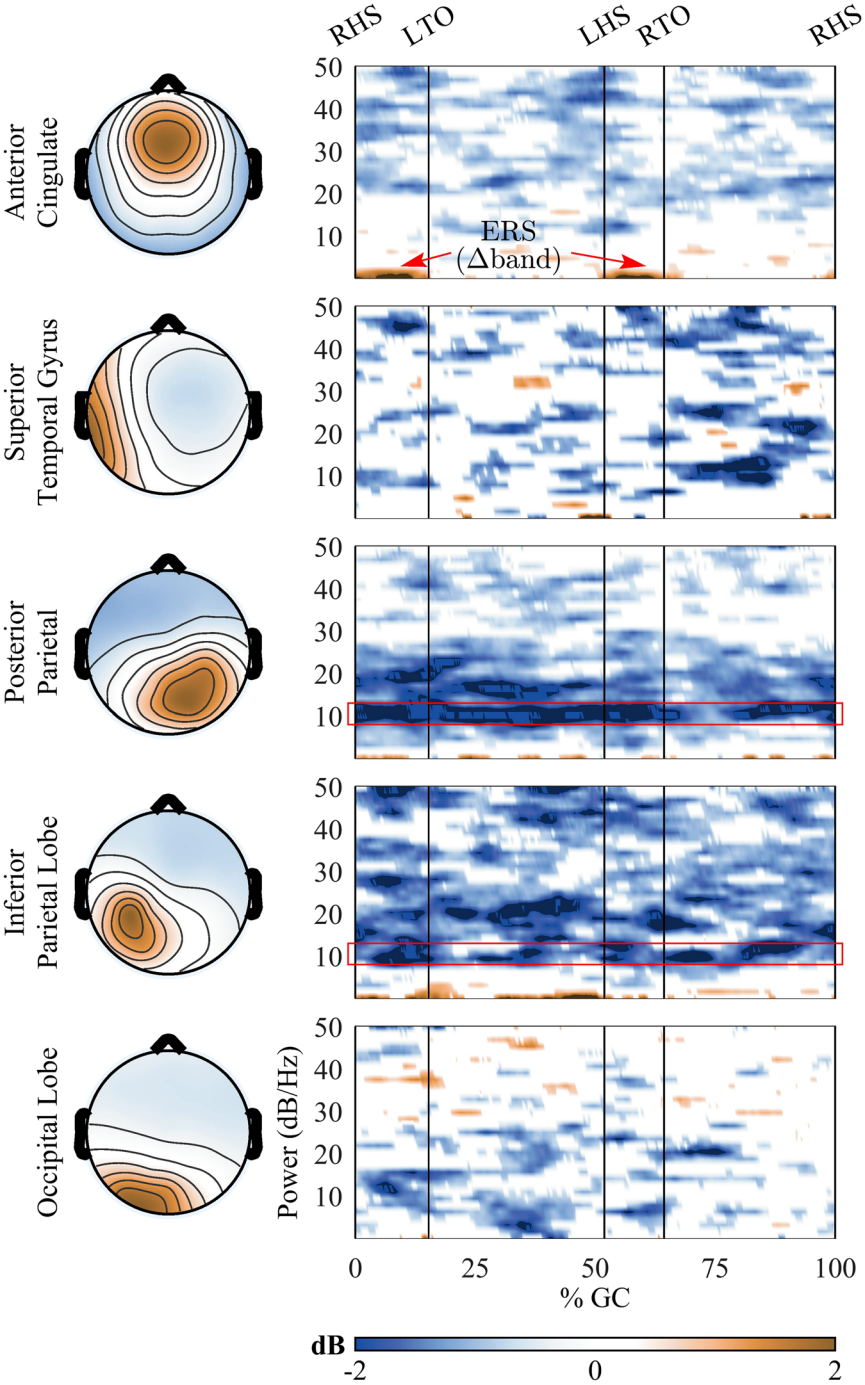
Spectral power difference between walking on a treadmill with and without BCI control of a walking avatar, *BCI-ctrl* and *Gonio-ctrl* phases, respectively. The red rectangles illustrate sustained ERD in the alpha/mu band. Time-frequency spectrograms were masked with non-significant differences set to 0 dB. Non-parametric bootstrapping technique with random shuffling was used for the comparison, significant level: 0.05.

We computed difference spectrograms between walking with and without BCI control of the walking avatar, *BCI-ctrl* and *Gonio-ctrl*, respectively. The significant differences in the spectrograms were obtained by using a non-parametric bootstrapping technique with random shuffling. Non-significant differences (α > 0.05) were set to 0 dB in Fig. 6. The spectral power in the α/µ band (8–13 Hz) substantially decreased (ERD) in the PPC and IPL regions during walking in the *BCI-ctrl* phase as compared to the *Gonio-ctrl* phase. The decreased power in the α/µ band was also sustained across the whole gait cycle. Significant decreases (ERD) were observed in the β band (14–30 Hz) band across all the clusters. However, the ERD in the β band was only observed intermittently throughout the gait cycle. The results from Fig. 6 also show significant increases (ERS) of spectral power in the low frequency bands (Δ: 0.1–3 Hz) in the ACC. Interestingly, this increased spectral power was more pronounced in the double support phase and early swing phase where the mismatch between the subjects’ gait patterns and the walking avatar was larger than in the stance phase (Fig. 6).

**Figure 7:**
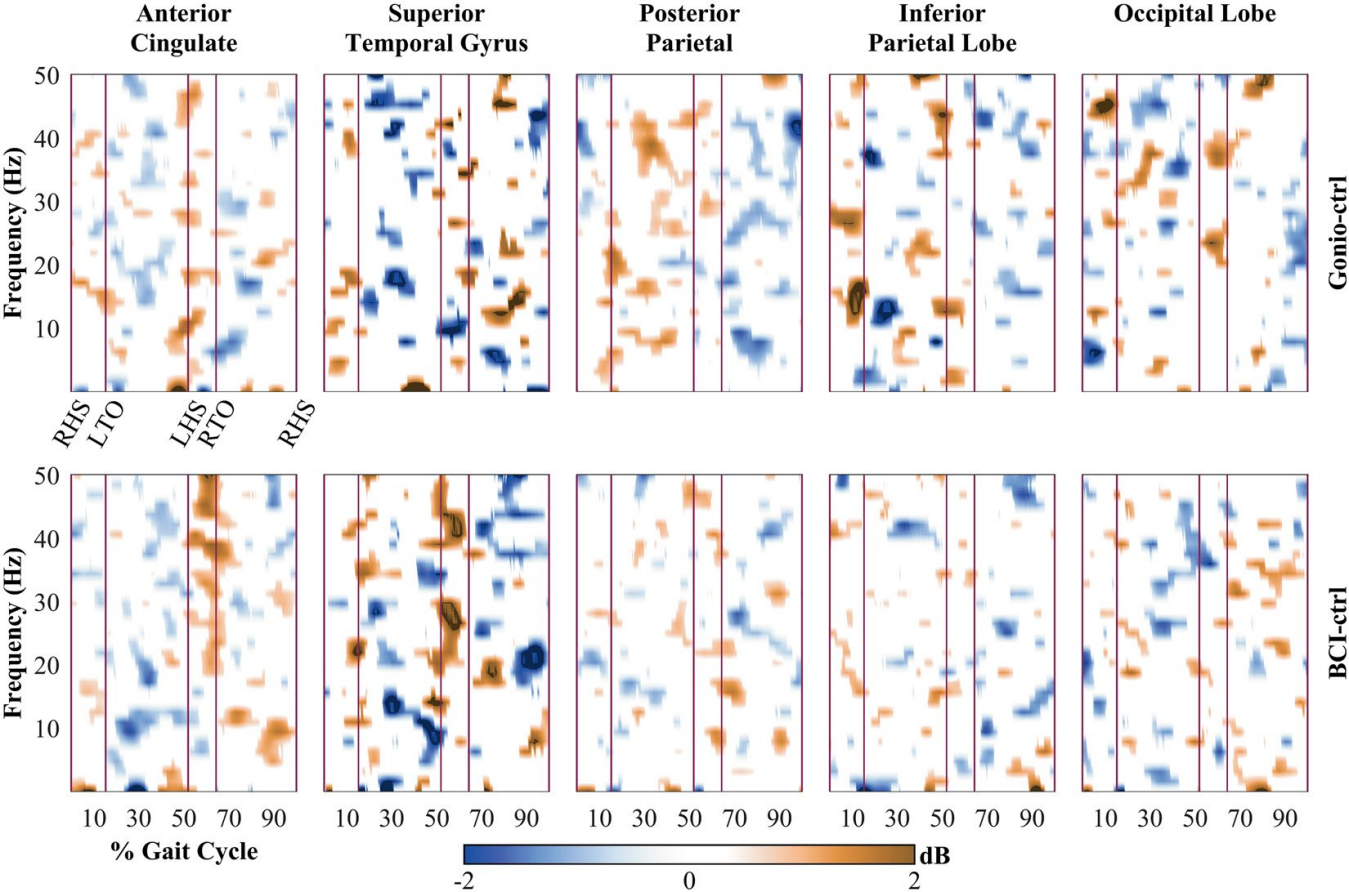
Event related spectral perturbations (ERSPs) for each cluster during treadmill walking without (*Gonio-ctrl* phase) and with BCI control of an avatar (*BCI-ctrl* phase). Significant ERSP values were identified using a bootstrapping technique with random shuffling and non-significant values were set to 0 dB. Significant level: 0.05.

We observed amplitude modulations relative to the mean gait cycle in the α/µ bands in the ACC, STG, and IPL during the *Gonio-ctrl* phase (Fig. 7). The β modulations in the STG region were enhanced when the participants walking with BCI control of the avatar (*BCI-ctrl* phase). Additionally, gait phase modulation in the β and low γ bands of ACC, and in the low γ band of STG were present during the *BCI-ctrl* phase. Figure 7 also shows greater ERSP values in *BCI-ctrl* phase in the ACC and STG during double leg support period right before the swing period of the right leg.

## Discussion

To our best knowledge, this study is the first to examine cortical involvement during treadmill walking while controlling a virtual avatar using a closed-loop EEG-based BCI-VR. Source localization using independent component analysis and dipole fitting revealed significant differences in cortical activity between walking with and without closed-loop BCI control. Specifically, we found sustained α/µ (8–13 Hz) suppression in the PPC and IPL regions, while less sustained but still significant decreases (ERD) were observed in the β band (14–30 Hz) band across all the clusters, indicating increased cortical involvement in the walking task with real-time brain control of a virtual avatar. Our results also showed significant increases in cortical activity in the low frequency (Δ bands) in the ACC area suggesting the possible benefit of using a closed-loop BCI-VR that recruits cortical network involved in error monitoring and motor learning. Additionally, we observed β suppression in the ACC, PPC, and IPL and the presence of low γ modulation in the ACC and STG. Such results indicate that our closed-loop BCI-VR system promotes voluntary control of human gait.

### Real-time closed-loop BCI-VR system to a walking avatar enhances cortical involvement in the gait task

Our results showed significant decreases in the α/µ and β bands of the ACC, STG, PPC, and IPL regions - as indicated by the area under the PSD curve (Fig. 5) and sustained desynchronization (ERD) in Fig. 6 - during the *BCI-ctrl* phase compared to the *Gonio-ctrl* phase. However, increased power in the low frequency (Δ bands) in the ACC region was observed in Fig. 6. Significant decreased power in the α/µ and β bands have been showed in recent studies when participants were actively involved in the walking tasks^7, 27^. Bulea *et al*. suggested that the decreases in power in the α/µ and β bands indicate increased cortical involvement when participants walked on an active treadmill or with increased gait speed. In the latter study, the decreased power in these bands were found in the sensorimotor areas when participants attempted to actively walk with a robotic assisted gait trainer as opposed to passively allowing the robotic system to move their legs. Furthermore, numerous studies have shown that decreased spectral power in these bands is correlated with higher neuronal activation due to increased task demand^30^, i.e. visual spatial attention and sensorimotor integration^31, 32^. Therefore, our results indicate that walking while controlling a virtual avatar using a real-time closed-loop BCI-VR system enhances cortical involvement in the gait task.

### Closed-loop BCI control of a walking avatar promotes cortical network activity involved in error monitoring and possibly adaptation learning

While spectral power in the α/µ and β bands decreases (desynchronize) with increasing task demands, the low frequency band power is known to increase (synchronize) in response to increasing task demands^33^. Previous studies also showed θ oscillations of EEG signals correlated with task difficulty^34^, error monitoring and learning processes^33, 35^, and memory and decision making^36^. Interestingly, a study from Womelsdorf *et al*. reported that local field potential (LFP) theta-activity in the Anterior Cingulate Cortex (ACC) of macaque monkeys holds the core functions in cognitive control processes and in error monitoring^37^. fMRI studies with human individuals also showed evidence for a direct relation between ACC activity and error detection and behavioral adjustment^38^, and the online monitoring of performance^39^. Interestingly in our study we found significant increased power in the low frequency (Δ bands) in the ACC region during walking with closed-loop BCI control (Fig. 6). Moreover, the difference is more pronounced in the double leg support and early swing phases. Results from ERSP analysis in Fig. 7 also show greater ERSP values in BCI-ctrl phase during the double leg support period just before the swing period of the right leg. The increases of ERSP values were found in the ACC and STG. Interestingly, the mismatch between the subjects’ gait pattern and the walking avatar is more obvious during the swing phase (Fig. 3). The changes of neural activities right before the swing period of the right leg observed in Figs 6 and 7 could be error-related responses from cortical networks to visual-motor feedback errors^40^. This suggests that using a closed-loop BCI-VR promotes cortical network activity involved in error monitoring and possibly adaptation learning.

### Action observation may differentially recruit the mirror neuron system (MNS) during BCI control of the walking avatar

We noted that during the closed-loop BCI control of the avatar, changes in cortical activity could reflect the conflict-solving between contrasting proprioceptive afference and visual afference during closed-loop BCI control of the avatar (i.e., a mismatch). Nevertheless, the ‘action observation network’ might be recruited during both *Gonio-* and *BCI-ctrl* conditions as avatar motion observation is critical for movement planning and adaptation. It has been postulated that the mirror neuron system (MNS) has evolved to understand other people’s actions, and during planning and performing self-made actions as in the present experiment^41^, where the MNS may also be recruited during closed-loop BCI control. Note that under the *BCI-ctrl* phase, the avatar’s motion represented the motor intentions inferred by the closed-loop BCI. Thus, the avatar performance, via the visual feedback, can then be compared with the motor intentions, which may lead to an error (error monitoring). This error can be used by the brain to improve BCI control. Indeed, our previous study showed that the subjects significantly improve their performance in closed-loop BCI control of the walking avatar using their brain signals across eight days of practices^21^.

### The changes in spectral content of cortical networks in the BCI control task may correlate with voluntary control of human gait

Voluntary control of movements is crucial for motor learning and physical rehabilitation^42, 43^. Our results suggest the possible benefits of using a closed-loop EEG-based BCI-VR system in inducing voluntary control of human gait. First, we observed significant decreases in the β spectral power and significant decreased power in the β band of ACC, PPC, and IPL (Figs 5 and 6). It is commonly accepted that the oscillations in the β band promote the current sensorimotor state or motor set^44^ while compromising processing related to new or voluntary movements^45–47^. A recent study from Seeber *et al*. also showed EEG β-suppression across the whole gait cycle during walking compared to standing^4^. Thus, the presence of β-ERD may suppress an inhibitory network and enable voluntary control of movements. Second, our results demonstrate that gait phase modulation in the low γ band was present in the ACC and STG regions while participants controlled the walking avatar using the closed-loop BCI-VR system (Fig. 7). Recent studies have shown that low γ amplitudes are modulated related to the gait phase^3, 4, 7^. Furthermore, the modulation in the low γ oscillations has been related to voluntary walking when subjects attempted to actively walk with a robotic trainer compared to passively allowing it move their legs^7^, or when they performed the task of active gait adjustment in a virtual environment^48^. A recent study from Bulea *et al*. also demonstrated that low γ band synchronization is enhanced in sensorimotor area and PPC while participants voluntarily tracked a target speed on an active treadmill^27^. These results are in line with our findings in this study that provides additional evidence that associates cortical activity with voluntary control of human gait. Our results also support the hypothesis that the sensorimotor system shifts its oscillations to the low γ band to plan and execute more complex movements^27^ in response to increasing motor task demands and attentions^49, 50^. We therefore conclude that our closed-loop EEG-based BCI-VR system promotes voluntary control of human gait. We also hypothesize that such a system is more immersive compared to walking with visual feedback using a VR system alone.

### Limitations and future work

Our current study demonstrated substantial changes in cortical activity when the subjects switched from open-loop (*Gonio-ctrl* phase) to closed-loop BCI-control of a walking avatar. The results further support previous studies suggesting that neural signals are highly dynamic^51, 52^ and neuroplasticity holds a critical role in developing robust neural decoder^53^. Neural decoders are typically trained offline by fitting neural signals against actual movements. This approach, however, ignores the neural dynamics from subjects when switching from open-loop to closed-loop BCI and typically results in decreased online performance. Based on previous studies^54, 55^ showing that closed-loop decoder adaptation (CLDA) yields performance improvement, we trained and updated the neural decoder at a fixed interval (one min). Although the decoding accuracy is promising, this study did not consider utilizing the information from cortical activity to optimize the neural decoder, i.e., optimal EEG channels for neural decoding of human gait have not been explored.

Future work has to examine if our closed-loop EEG-based BCI-VR could be deployed during observational therapy in post-stroke rehabilitation, which has been shown to be beneficial to the rehabilitation process^56–58^. In typical observational therapy, persons with stroke observe a video clip or other people demonstrating a movement before they try it themselves. Reports suggest that patients undergoing observational therapy show significant improvement in functional assessment before and after the treatment^57^. Based on the results from this study and our previous findings^21^ suggesting the feasibility of using a closed-loop BCI-VR with visuomotor perturbations to induce cortical adaptations, we hypothesize that our system could be clinically meaningful in a neurorehabilitation context.

## Conclusions

Whereas previous studies have analyzed cortical activity during typical treadmill walking, walking with a robotic assisted gait trainer, or with varying gait speeds using an active treadmill, this study is the first to examine cortical involvement during treadmill walking while controlling a virtual avatar using a closed-loop EEG-based BCI-VR. Interestingly, we observed significant differences in cortical activity between walking with and without closed-loop BCI control. In particular, we found sustained α/µ suppression in the PPC and IPL regions and significant decreases (ERD) in the β band, indicating increased cortical involvement in the walking task with real-time control of a virtual avatar using a closed-loop BCI-VR system. Our results also showed significant increased cortical activity in the low frequency (Δ bands) in the ACC area suggesting the possible benefit of using a closed-loop BCI-VR that recruits cortical network involved in error monitoring and motor learning. Additionally, we observed β suppression in the ACC, PPC, and IPL and the presence of low γ modulation in the ACC and STG. Such results indicate that our closed-loop BCI-VR system promotes voluntary control of human gait. Thus, the current study demonstrated the feasibility of using a closed-loop EEG-based BCI for controlling a walking avatar, promoting cortical engagement, and monitoring cortical activity. Our closed-loop BCI-VR system may be relevant for neurological gait rehabilitation as a clinical tool for post-stroke physical training and clinical assessment. Finally, our system may also help to better understand cortical dynamics during walking with a closed-loop BCI system.

## Electronic supplementary material

Supplementary  Information
