# Supplementary material for: Real-time EEG-based brain-computer interface to a virtual avatar enhances cortical involvement in human treadmill walking
Source: Sci Rep. 2017 Aug 21;7:8895. doi: 10.1038/s41598-017-09187-0 (PMC5567182; doi:10.1038/s41598-017-09187-0)
Supplement: Supplementary file 1 — Supplementary Information [file 41598_2017_9187_MOESM1_ESM.pdf]

Supplementary Material

Real-time EEG-based brain-computer interface to a virtual avatar enhances cortical involvement in human treadmill walking

Trieu Phat Luu*, Sho Nakagome, Yongtian He, and Jose L. Contreras-Vidal

Noninvasive Brain-Machine Interface System Laboratory,

Dept. of Electrical and Computer Engineering,

University of Houston, Houston, TX 77004, USA.

Correspondence:

Trieu Phat Luu. Email: ptluu2@central.uh.edu

Conflict of Interest: The authors have no conflicts of interest.

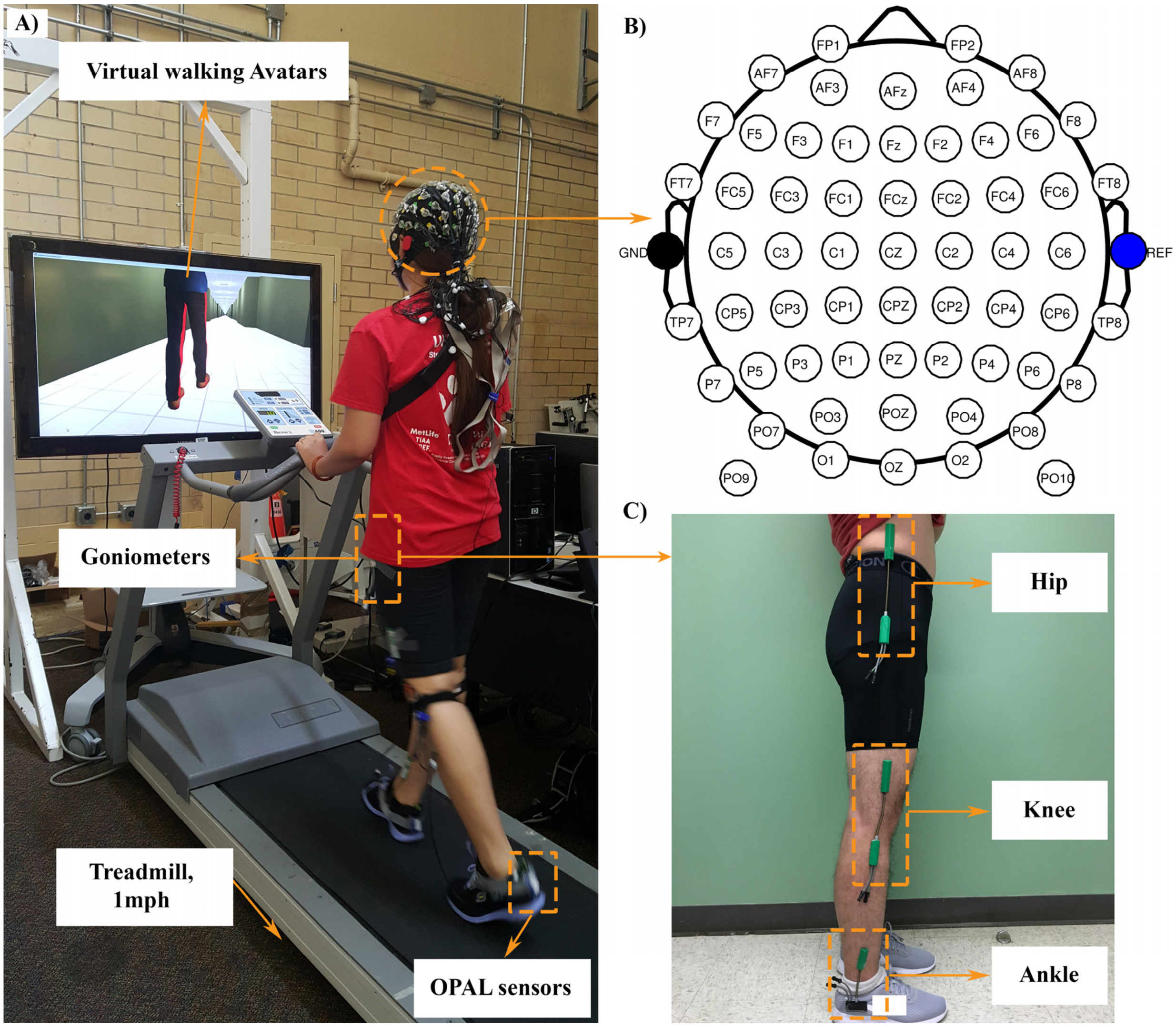

Supplementary Figure 1: A) Experimental setup in this study. Each subject was instrumented with non-invasive EEG cap, three goniometer sensors to measure lower limb joint angles (hip, knee, and ankle) on each side, and three OPAL sensors placed at the forehead, left, and right heels. The treadmill speed was gradually increased and fixed at 1 mile per hour (mph). The subjects was instructed to look at the virtual walking avatar displayed on a monitor during the entire trial. B) EEG cap layout used in this study. The channel layout was modified from the standard EEG cap setup. Ground (GND) and reference (REF) channels were placed on the left and right earlobe (A1 and A2), respectively. T7 and T8 channels were moved to FCz and AFz, respectively. FT9, FT10, TP9 and TP10 were used for electrooculography (EOG) to capture eye blinks and eye movements. The two main reasons for the modification are: 1) GND and REF channels in the standard EEG cap layout are very close to the motor cortex, and 2) EOG sensors were required in our real-time artifact removal algorithm. C) Example of goniometer sensor setup to measure right hip, knee, and ankle joint angles.
